# Loss of 11p11 is a frequent and early event in sporadic nonfunctioning pancreatic neuroendocrine neoplasms

**DOI:** 10.3892/or.2014.3328

**Published:** 2014-07-11

**Authors:** SVEN-PETTER HAUGVIK, LUDMILA GORUNOVA, LISBETH HAUGOM, ANNE METTE EIBAK, IVAR PRYDZ GLADHAUG, SVERRE HEIM, FRANCESCA MICCI

**Affiliations:** 1 Department of Hepato-Pancreato-Biliary Surgery, Rikshospitalet, Oslo University Hospital, 0372 Oslo, Norway; 2 Institute of Clinical Medicine, University of Oslo, 0372 Oslo, Norway; 3 Section for Cancer Cytogenetics, Institute for Cancer Genetics and Informatics, The Norwegian Radium Hospital, Oslo University Hospital, 0372 Oslo, Norway; 4 Centre for Cancer Biomedicine, University of Oslo, 0372 Oslo, Norway

**Keywords:** pancreatic neuroendocrine neoplasm, pancreas, NET, NEN, karyotyping, CGH, genomic imbalances, cell proliferation

## Abstract

The pathogenesis of sporadic pancreatic neuroendocrine neoplasms (PNENs) is poorly understood. To gain insight into the genetic mechanisms underlying this tumor entity, we performed genome-wide screening of 16 surgical specimens from 15 patients with sporadic PNEN, combining G-band karyotyping and high resolution comparative genomic hybridization (HR-CGH). G-banding revealed abnormal karyotypes in 2 of 10 tumor samples analyzed. DNA copy number changes were detected in 13 samples, whereas three tumors showed a balanced genome. Gains were more frequent than losses in the nonfunctioning tumors (n=13). Common gains were scored at 5p12–13, 4q13–24, 5p15, 5q11–31, and 9q21–22. Common losses were scored at 11p11, 11p14–15, 11q23, 11p12–13, and 11q22. The average number of copy aberrations (ANCA index) was 12 for 13 nonfunctioning primary tumors, 4.8 for the nonfunctioning tumors with low Ki-67 (≥5%), 21.2 for the tumors with high Ki-67 (<5%), 2.5 for small tumors (<3.5 cm), and 17.8 for large tumors (≥3.5 cm). There was a statistically significant difference in the ANCA index between the groups defined by Ki-67 and tumor size. Nonfunctioning tumors with low Ki-67, no distant metastasis and small size had few aberrations detected by HR-CGH, but frequent loss of material from chromosomal band 11p11. The present study indicates the existence of distinct cytogenetic patterns in sporadic nonfunctioning PNEN. Loss of chromosomal band 11p11 might indicate a primary pathogenetic event in these tumors.

## Introduction

Pancreatic neuroendocrine neoplasms (PNENs) represent 2–4% of all clinically detected pancreatic tumors (1), with an incidence of 2–3 per million based on data from the US and Norway (2,3). PNENs are generally classified according to their tumor-node-metastasis (TNM) pattern (4), grading as it emerges from histopathological findings (5,6), and biologic behavior, which depends on the presence of clinical symptoms caused by abnormal hormone secretion.

At present, clinical management of patients with PNEN is largely based on TNM stage and grading. However, the malignant potential among PNENs at the same TNM stage and of the same grade may vary considerably. A more precise classification of PNEN based on the tumors’ pathogenetic characteristics might predict their inherent aggressiveness better and further knowledge of the molecular pathology of this disease might serve as a starting point for novel therapies directed specifically against the primary event behind tumorigenesis.

PNEN, as cancer in general, is the phenotypic result of the acquisition of one or more genomic change(s) taking place at the chromosomal and/or gene level. Thus, screening of the whole tumor genome is a natural starting point when trying to understand the pathogenetic mechanisms behind tumor development (7). Although studies of PNEN using comparative genomic hybridization (CGH) have been performed (8), the results thus obtained have not been correlated with karyotyping and cell proliferation data.

The Mitelman database on chromosome aberrations and gene fusions in cancer (9) reports seven PNENs with karyotypic aberrations, but no common chromosomal abnormalities (10–12). Thus, knowledge regarding the chromosomal characteristics of this type of cancer is clearly insufficient. Information on genomic imbalances in nonfunctioning (i.e. without hormone production) PNEN detected by CGH is limited to 54 cases (13–16). Common genomic imbalances involved gains of 7q, 17q, and 20q and losses of 6q, 11p, and 11q. The available CGH-data on PNENs were obtained on small and heterogeneous series of tumors and it is therefore difficult to generalize the findings (8).

In the present study, we sought to gain more information regarding the genomic rearrangements in PNENs by performing karyotyping of G-banded chromosomes and high resolution CGH (HR-CGH) analyses on a series of sporadic PNENs.

## Materials and methods

### Tumor samples and clinicopathological data

The tumor samples were obtained from a prospective series collected between April 2011 and January 2013, at the Department of Hepato-Pancreato-Biliary Surgery, Rikshospitalet, Oslo University Hospital. Sixteen specimens from 15 patients were included in the study (Table I); 15 stemmed from the pancreas, whereas one sample was of concurrent metastatic tissue in the liver of the same patient (case 15b). The patients had a median age of 57 years (range, 30–78). The tumors were diagnosed by an experienced pathologist according to the World Health Organization 2010 classification (5). Tumors were classified as functioning if they caused clinical symptoms due to hormone secretion and immunohistochemistry confirmed hormone overproduction. Cases 10 and 15 had functioning tumors (insulinoma). Thirteen patients had nonfunctioning tumors, indicating that they did not have clinical symptoms due to hormonal secretion. In order to allow for subdivision of the tumor samples analyzed, the following tumor characteristics were registered: degree of cell proliferation as indicated by the value of Ki-67 (< or ≥5%), the presence/absence of synchronous distant metastases, and the size of the primary tumor (< or ≥3.5 cm). We used a Ki-67 cut-off value of 5% as this is a known prognostic predictor (17,18). The cut-off value of the size of the primary tumor was chosen in such a way that an equal amount of analyzed samples were below and above this value.

The study was approved by the Regional Ethics Committee (project number: 2011/497A and 2011/1945D). Informed consent was obtained from all patients.

### G-banding and karyotyping

Representative fresh samples from 10 PNENs were sent to the Section for Cancer Cytogenetics immediately after the operation. The samples were disaggregated mechanically and enzymatically using collagenase Type II (Worthington, Freehold, NJ, USA). The resulting cells and cell clumps were seeded into tissue culture flasks and cultured in DMEM/Ham’s F12 medium supplemented with 10% fetal bovine serum (FBS), 1% penicillin/streptomycin, 1% ITS+ Premix, 20 ng/ml epidermal growth factor (both from BD Biosciences, Bedford, MA, USA). After 7–10 days, the cultures were harvested as described by Mandahl (19). Chromosome preparations were G-banded using Wright stain and karyotyped according to the ISCN (2009).

### High Resolution Comparative Genomic Hybridization (HR-CGH)

Representative fresh-frozen tissues stored at −80°C in a biobank were used for molecular cytogenetic analysis. DNA from 16 tumor samples was isolated using the MagAttract DNA Mini M48 kit (Qiagen, Valencia, CA, USA). HR-CGH was performed according to our modifications of the standard procedure (20–22). Chromosomes were karyotyped based on their inverted DAPI appearance and the relative hybridization signal intensity was determined along each chromosome. On average, 10–15 metaphases were analyzed. The description of the CGH copy number changes was based on the recommendations of the ISCN (2009).

## Results

Of the 10 cytogenetically analyzed tumor samples (Table II), eight showed normal karyotype whereas two were abnormal (cases 9 and 10). Case 9 had an extra chromosome 12 as the only clonal aberration in an otherwise incomplete karyotypic description, while case 10 showed a near tetraploid genome that could not be further described (Table II).

**Figure 1:**
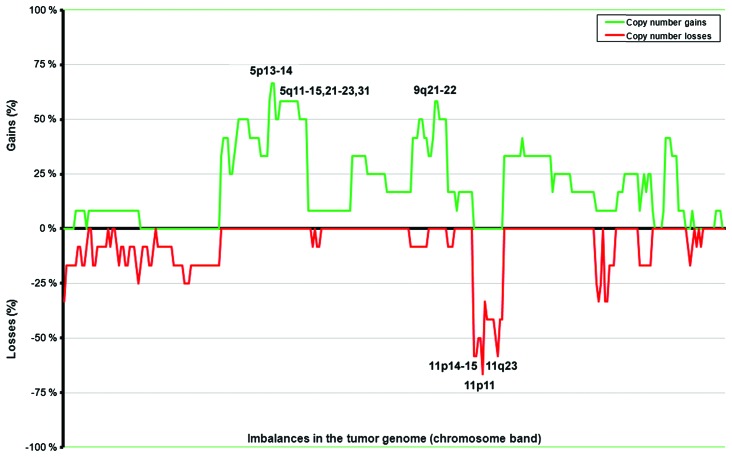
Frequencies of chromosomal gains and losses detected by HR-CGH in 13 patients with sporadic nonfunctioning PNEN.

The HR-CGH analysis provided informative results in all 16 tumors analyzed. In 13 tumors, DNA copy number changes were detected, whereas three tumors (case 1, 11 and 15) showed a balanced genome (Table II). Of the 16 tumor samples, 13 were nonfunctioning tumors. In general, gains were more frequent than losses in these PNENs, but no amplification was scored. Commonly gained regions in the nonfunctioning tumors were found at 5p12–13 (in seven out of 11 tumors with abnormalities; 64%) and at 4q13–24, 5p15, 5q11–31, and 9q21–22 (in six tumors; 55%; Fig. 1). Losses were scored at 11p11 (in eight tumors out of 11 with imbalances; 73%) followed by 11p14–15 and 11q23 (seven tumors; 64%), and 11p12–13 and 11q22 (six tumors; 55%). The average number of copy aberrations (ANCA index) was 12 for the 13 nonfunctioning primary tumors.

We further subdivided the nonfunctioning tumors according to the degree of cell proliferation as indicated by the value of Ki-67 (< or ≥5%), the presence/absence of synchronous distant metastases, and the size of the primary tumor (< or ≥3.5 cm).

**Figure 2:**
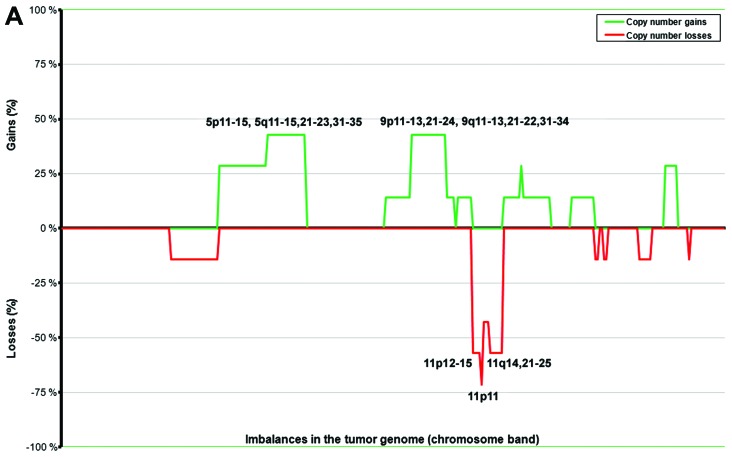

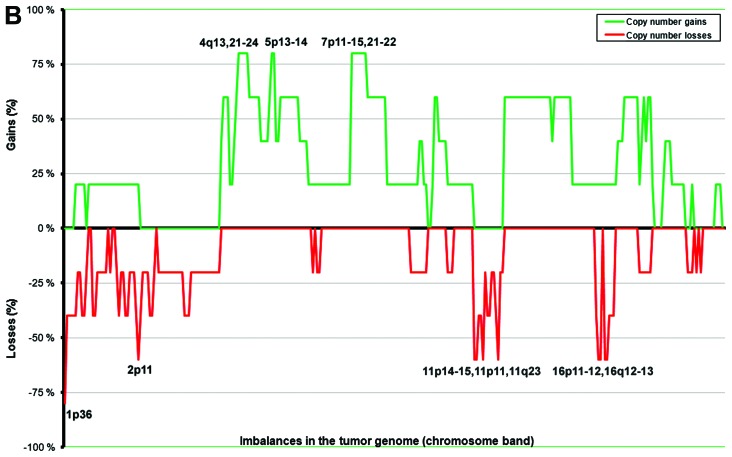
Frequencies of chromosomal gains and losses detected by HR-CGH in patients with sporadic nonfunctioning PNEN and (A) Ki-67 <5% (n=8), or (B) Ki-67 ≥5% (n=5).

The ANCA index for the tumors with low Ki-67 was 4.83, whereas the tumors with high Ki-67 had an ANCA index of 21.2. An overview of the genomic imbalances and the specific values of the parameters used is presented in Tables I and II. The tumors with high Ki-67 values (≥5%) showed a more complex picture of imbalances compared to those with low Ki-67 (Fig. 2), which showed fewer imbalances or a balanced genome (two cases). The subgroup with high Ki-67 showed frequent gains at 4q13–24, 5p13–14, and 7p11–22 (in four out of five tumors with imbalances; 80%), followed by 4p13–15, 4q25–31, 5q11–31, 7q11–36, 9q21–22, 12p, 12q, 13q, 14q12–33, 17q, 18q12, and 18q22–23 (in three tumors; 60%). Losses were present at 1p36 (five tumors with imbalances; 80%) followed by 2p11, 11p14–15, 11p11, 11q23, 16p11–12, and 16q12–13 (four tumors; 60%). The subgroup with low Ki-67 showed gains of one copy of each of chromosomes 5 and 9 in three tumors out of six with imbalances (50%). Losses were scored at 11p11 (83%), 11p12–15 and 11q14–25 (67%), and 11q11–13 (50%).

The tumors with synchronous distant metastases were the same as those with Ki-67 ≥5%. Therefore, the imbalances as well as the ANCA index are the same in the two subgroups.

**Figure 3:**
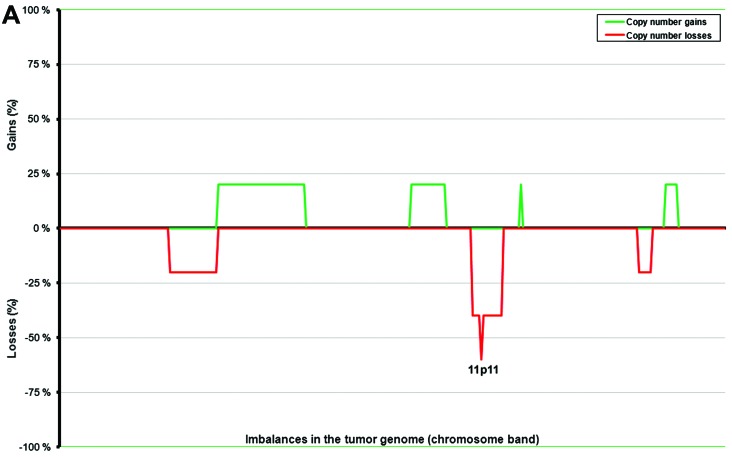

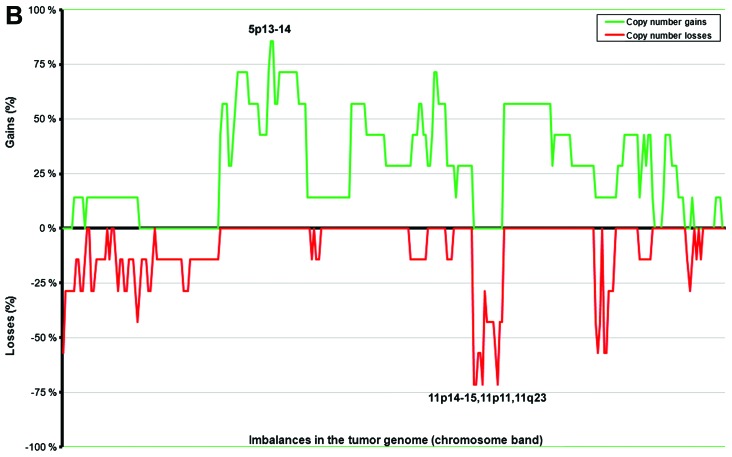
Frequencies of chromosomal gains and losses detected by HR-CGH in patients with sporadic nonfunctioning PNEN and (A) primary tumor diameter <3.5 cm (n=5), or (B) primary tumor diameter ≥3.5 cm (n=8).

In general, the tumors with a diameter <3.5 cm (n=5, range 0.7–2.5 cm) showed fewer imbalances than those with a diameter ≥3.5 cm (n=8, range 3.5–10.0 cm) (Fig. 3). The ANCA index for the first subgroup of tumors (size <3.5 cm) was 2.5, whereas for the latter (size ≥3.5 cm) it was 17.85. To some extent, the imbalances also affected different genomic regions in the two subgroups. More precisely, the larger tumors showed common gains of 5p13–14 (in six out of seven tumors with imbalances; 86%) followed by 4q13–24, 5p15, 5q11–31, and 9q21–22 (five tumors; 71%), and 4p13–15, 4q12, 4q25–31, 5p12, 5q32–35, 7p, 9p13–21, 9q31–34, 12p, 12q, and 13q (57%). Losses were scored more frequently at 11p14–15, 11p11, and 11q23 (five out of seven tumors with imbalances; 71%) followed by 1p36, 11p12–13, 11q22, 11q24, 16p12, and 16q12–13 (57% of tumors with imbalances) (Fig. 2A). The smaller tumors showed losses from chromosome 11 as the only common imbalance; more precisely, 11p11 was lost in three out of four tumors with imbalances (75%), whereas chromosomal regions 11p12–15 and 11q were lost in 50% of the tumors with imbalances.

To assess the values scored for each subgroup, we used the non-parametric Mann-Whitney U test which showed a significant difference within all subgroups. Specifically, we found a statistically significant difference in the amount of copy number changes in the group with low Ki-67 value compared to the group with high Ki-67 value (P=0.017); the same statistically significant difference was found for the number of aberrations found in the tumors with synchronous distant metastases and the group without. Furthermore, the subgroups of tumors smaller and larger than 3.5 cm also showed a statistically significant difference in the number of changes (P=0.018) (Table III).

The two cases of primary insulinoma, the only functioning tumors in our series, showed gains of the entire chromosomes 5, 9, and 20 (case 10) and a balanced genome (case 15a). The liver metastasis from the latter case (15b) showed gains at 2q22–24,4q13, and 13q and losses at 1p, 3, and 9q.

## Discussion

Pronounced variability from case to case has made research on pancreatic neuroendocrine neoplasms (PNENs) demanding. In order to improve our understanding of the relationship between clinical behavior, histopathology and the acquired genetics of this tumor entity, systematic analysis of defined series of PNENs is required.

Only two of the ten tumors sent for cytogenetic analysis showed clonal chromosome abnormalities. In both cases, the karyotypic description was incomplete and the number of abnormal cells constituted a minority. However, case 9 showed an extra chromosome 12 by karyotyping, which was also found gained, among other imbalances, by HR-CGH, indicating that both techniques identified the same, or at least related, abnormal clones. Karyotyping of tumors requires culturing of neoplastic parenchyma cells *in vitro*. It appears that pancreatic neuroendocrine tumor cells do not divide well under laboratory conditions and this may account for the severely limited cytogenetic information of PNENs hitherto reported in the literature with only seven karyotypical abnormal cases in three studies (10–12).

Investigations of genomic imbalances by means of CGH are easier in this regard inasmuch as it does not require the neoplastic cells to enter mitosis. Most of the available data from CGH studies of PNENs were obtained on small and heterogeneous series. Moreover, different tumor classifications were used by the investigators, making a comparative analysis of different PNEN subtypes difficult. We used HR-CGH to screen the genomes of 16 PNEN samples for imbalances. All tumors yielded informative results with 13 showing imbalances. The use of dynamic standard reference intervals (D-SRI), which represent a ‘normal’ ratio profile that takes into account the amount of variation detected in negative controls for each chromosome band, provided more objective and sensitive scoring criteria than fixed thresholds and, consequently, a higher resolution. In our series, most patients presented with nonfunctioning tumors (n=13). In these, the most frequent imbalance (loss) was scored for chromosome 11. Loss of chromosome 11 has been previously reported (13,23). In our study, 11p11 was found lost in 73% of the nonfunctioning tumors with imbalances, followed by 11p14–15 and 11q23 (64% of the tumors), and 11p12–13 and 11q22 in 55%. Further studies at the gene level may provide information as to the possible presence of tumor suppressor genes of importance in PNEN tumorigenesis and/or progression at this location. The most frequently gained region was 5p12–13 in the nonfunctioning tumors, and overall gains were present in 64% of the nonfunctioning tumors with imbalances. Previous studies have shown frequent gain of chromosome 5 (13,23); however, this is the first time that a more detailed map of specifically gained bands was identified. The finding indicates the location of possible oncogenes active in this tumor type. The second most frequently gained regions were 4q13–14 and 9q21–22, both found altered in 55% of the abnormal nonfunctioning tumors. Previous reports have suggested frequent gains of chromosome arms 4q and 9q (13,16,23), and we were able to restrict the commonly gained areas to two chromosomal bands.

We further subdivided the nonfunctioning tumors with regard to the degree of cell proliferation as measured by the value of Ki-67, the presence of synchronous distant metastases, and the size of the primary tumor. The number of copy number changes, indicated by the ANCA index, was 4.8 for tumors with low Ki-67 (≥5%) and 21.2 for the group with high Ki-67 (<5%). The same values were also found for tumors with and without synchronous distant metastases, and 2.5 for small tumors (<3.5 cm) and 17.8 for larger tumors (≥3.5 cm).

Notably, the three subgroups of nonfunctioning tumors with low values of Ki-67, no distant metastasis and small size, all had in common not only the fact that they showed few aberrations, but also frequent loss of material from chromosomal band 11p11 (in 83, 83, and 75% of the abnormal tumors, respectively). This suggests that loss of 11p11 may be a particularly important, possibly primary, event in the tumorigenesis of sporadic nonfunctioning PNENs. The large tumors with high Ki-67 and distant metastases all showed additional imbalances in their genome probably acquired during tumor progression. To know the primary event of tumorigenesis, the *conditio sine qua non* for the tumor to develop, is fundamental for the identification of potential tailor-made treatment of these patients that targets only the abnormal cells. Furthermore, knowing the specific aberration is also a prerequisite for a correct pathogenetic classification of the tumor. The study of the above parameters is a step in the pathogenetic classification, but clearly we are still far from being able to establish what constitutes the primary molecular events during tumorigenesis.

The only functioning tumor (an insulinoma) with imbalances in our series (case 10) showed gains of one copy of chromosomes 5, 9 and 20. Gain of chromosome 9q is already known as a frequent aberration in both benign and malignant insulinomas (24), and gain of chromosomal band 9q34 has previously been described as an early event in insulinomas (14). The other abnormal insulinoma sample was a liver metastasis (case 15b) that showed gains at 2q22–24,4q13, and 13q and losses at 1p, 3 and 9q13–34 while the primary tumor (case 15a) showed a normal, balanced profile. Thus, it seems that chromosomal instability increased from the primary to the metastatic lesion. Jonkers *et al* found that chromosome 6q losses and 12q, 14q and 17pq gains are strongly associated with metastatic insulinoma (24). Floridia *et al* found that allelic loss of 22q correlated with distant metastasis (13). Our case did not confirm these findings. However, data from only one patient are too scarce to draw any conclusions in this regard.

## Figures and Tables

**Table I:** Clinicopathological characteristics of 15 patients with sporadic PNENs.

Case no. (biobank)	Gender/Age (years)	Tumor location	Tumor diameter (cm)	Clinical behavior	Ki-67 (%)	ENETS TNM^a^
1 (5)	m/66	Tail	4.0	Nf	0.8	T2N0M0
2 (10)	m/73	Tail	3.6	Nf	12.7	T2N1M1
3 (18)	m/52	Tail	10.0	Nf	32.6	T3N1M1
4 (19)	f/78	Tail	2.1	Nf	2.3	T2N0M0
5 (26)	m/68	Tail	0.7	Nf	0.8	T1NxM0
6 (27)	f/57	Head	2.5	Nf	1.7	T2N0M0
7 (31)	m/57	Head/body/tail	10.0	Nf	13.0	T4N0M1
8 (36)	f/33	Tail	1.5	Nf	1.0	T1N0M0
9 (37)	f/60	Tail	8.8	Nf	12.4	T3N0M1
10 (42)	f/37	Tail	1.3	F (insulinoma)	6.3	T1N0M0
11 (45)	m/40	Tail	1.2	Nf	1.6	T1N0M0
12 (47)	f/67	Body	3.5	Nf	1.3	T2N0M0
13 (54)	f/61	Tail	4.0	Nf	9.1	T2N0M1
14 (56)	f/51	Head/body/tail	5.0	Nf	2.1	T3N0M0
15a (59T)	m/30	Tail	1.5	F (insulinoma)	9.3	T1N1M1
15b (59L)	m/30	Liver metastasis	2.5	F (insulinoma)	26.0	NA

PNENs, pancreatic neuroendocrine neoplasms; m, male; f, female; Nf, nonfunctioning; f, functioning; NA, not applicable.

a ENETS TNM Classification 2010 (4).

**Table II:** Genomic alteration detected in the PNENs examined by karyotyping and HR-CGH.

Case no. (biobank)	Karyotype	CGH imbalances
1 (5)	-	No imbalances
2 (10)	-	rev ish enh(4q13,4q21q24),dim(1p,1q21q32,2p,2q11q22,2q23,2q31q35,2q36q37, 6p12p21,11p11p14,11q12q13,11q22q23,16p,16q12q23)
3 (18)	-	rev ish enh(1p12p31,1q,2p,5p,5q11q31,6,7,8,10,12,14,16,17,19p13,20p,21), dim(Xp22,1p31pter,3,9p,11,18)
4 (19)	-	rev ish dim(11,18)
5 (26)	46,XY[20]	rev ish dim(11)
6 (27)	46,XX[15]	rev ish dim(3)
7 (31)	46,XY[10]	rev ish enh(Xp22,Xq26q28,4p13p15,4q,5p13p14,5q,7,9p13p21,9q21qter,12p11p13, 12q,13,14q12q24,14q31q32,17q,18q,19q13),dim(1p36,2p11p12,3p13p21,8q24,11p1 4p15, 11q23,16p11p12,16q12q13)
8 (36)	46,XX[15]	rev ish enh(4,5,9,12q21,20),dim(11p11)
9 (37)	47~48,XX,+12,inc [cp 5]/46,XX[3]	rev ish enh(4p13pter,4q,5p13pter,7,9q21qter,12p11pter,12q,13,14,15,17,18p11,18q, 20p11pter,20q11qter), dim(Xp11,Xq12,1p36,1p12p13,1q21q22,1q42,2p24,2p21,2p16, 2p11,2q11,2q21q22,6p23,10p13p15,11p15,11p11,16p11p13,16q12q23,22q12q13)
10 (42)	81~87,inc[3]/46,XX[22]	rev ish enh(5,9,20)
11 (45)	46,XY[25]	No imbalances
12 (47)	46,XX[25]	rev ish enh(4,5,8,9,10p12pter,10q,12,13,20p,20q11q13),dim(11p,11q14qter,16p12p13, 16q12q13,22q13)
13 (54)	46,XX[25]	rev ish enh(4p,4q12q31,5p,5q,7p,9p,9q13q22,13q,18q12,18q22q23)
14 (56)	46,XX[25]	rev ish enh(5,9,15),dim(11)
15a/59T	-	No imbalances
15b/59L	-	rev ish enh(2q22q24,4q13,13q),dim(1p,3,9q13q34)

PNENs, pancreatic neuroendocrine neoplasms.

**Table III:** Average number of copy aberrations (ANCA index) in subgroups of cell proliferation (Ki-67), status of metastatic disease and tumor size in 13 patients with sporadic nonfunctioning PNEN.

	Ki-67		Distant metastasis		Primary tumor diameter (cm)	
			
	<5%	≥5%	yes	no	<3.5	≥3.5
ANCA index	4.83	21.2	4.83	21.2	2.5	17.85
U-test^a^ (P-value)	0.017		0.018		0.018	

a Mann-Whitney U test.

PNEN, pancreatic neuroendocrine neoplasm.
